# Dissociable pupil-linked arousal during overt and inner speech

**DOI:** 10.1016/j.isci.2026.117825

**Published:** 2026-10-09

**Authors:** Ryuta Kuwamizu, Ryoichi Watanabe, Nozomi Yamamoto, Kota Otani, Yusuke Moriguchi

**Affiliations:** 1 Institute of Health and Sport Sciences, University of Tsukuba, Tsukuba, Ibaraki 305-8574, Japan; 2 Graduate School of Letters, Kyoto University, Kyoto 606-8501, Japan; 3 Research Organization of Open Innovation and Collaboration, Ritsumeikan University, Ibaraki, Osaka 567-8570, Japan; 4 Graduate School of Human Sciences, The University of Osaka, Suita, Osaka 565-0871, Japan; 5 Japan Society for the Promotion of Science, Tokyo 102-0083, Japan; 6 Graduate School of Sociology, Ritsumeikan University, Kyoto 603-8577, Japan

**Keywords:** pupillometry, covert speech, arousal system, vocalization, mental counting

## Abstract

Inner (covert) speech, the silent production of language in one’s mind, plays a central role in human cognition and engages speech-related cortical networks partly overlapping with those for overt speech. Because cortical language processing unfolds within arousal states set by the ascending systems, we asked whether inner and overt speech are accompanied by similar pupil-linked arousal patterns. Using pupillometry across three counting-task experiments, we found a dissociation between inner and overt speech. While overt speech consistently elicited pupil dilation across conditions, inner speech produced a variable dilation profile relative to baseline: pupil constriction (simple counting), no deviation (noun-phrase counting), and clear dilation (step counting; high cognitive load). Importantly, pupil size during inner speech remained smaller than that during overt speech despite task-dependent dynamics. These findings suggest that task-elicited inner speech is accompanied by lower pupil-linked arousal than overt speech, adding a state-level physiological dimension to cortical accounts of verbal processing.

## Highlights

• A matched pupillometry paradigm compares inner and overt speech
• Overt speech consistently elicits pupil dilation across counting tasks
• Inner-speech pupil dynamics vary by task but remain below overt-speech responses
• Findings support a lower-arousal account of task-elicited inner speech

## Graphical abstract

**Figure:**
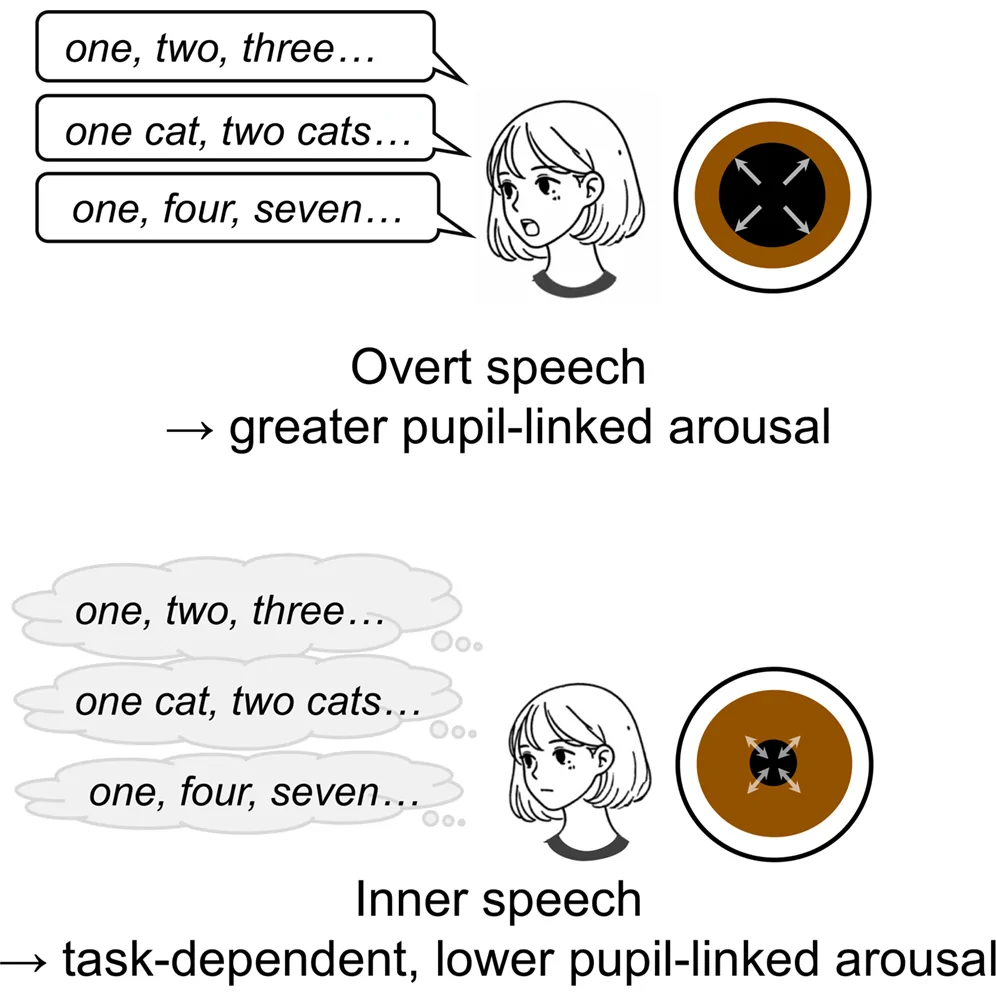

## Teaser

Natural sciences; Biological sciences; Neuroscience; Systems neuroscience; Cognitive neuroscience

## Introduction

There is an old saying that “the eyes speak as much as the mouth.” But do they speak the same language when we talk aloud versus when we talk silently to ourselves?

Inner speech, the act of speaking silently in one’s head, is a key cognitive tool for human thought and self-regulation.^1^ Neuroimaging studies over the past two to three decades suggest that inner speech, or covert speech, draws on a fronto-temporal speech-production network that partly overlaps with, but is not identical to, the network engaged during overt speech, including left inferior frontal, supplementary motor, insular, and superior temporal regions.^2^^,^^3^^,^^4^^,^^5^ The overlap is neither complete nor uniform in direction; depending on the region examined and the task used to elicit it, inner speech can produce weaker, comparable, or even greater activation than overt speech, with primary motor and auditory regions typically less engaged during covert production and regions associated with monitoring, inhibition, and preparation sometimes being more engaged.^1^^,^^6^^,^^7^ These observations have motivated motor-simulation and predictive-monitoring accounts, in which inner speech involves covert engagement of the speech-production system, with an efference copy of the planned utterance used to predict its auditory and somatosensory consequences.^8^^,^^9^ Such accounts, however, are now understood to capture only one component of a more heterogeneous phenomenon, rather than a complete characterization of inner speech.^3^ Inner speech itself takes multiple forms—condensed or expanded, monologic or dialogic, deliberate or spontaneous, and experienced in one’s own or another’s voice—that appear to rely on partially distinct neural systems,^4^^,^^10^ with dialogic inner speech, for instance, recruiting a broader bilateral network including superior temporal, posterior cingulate/precuneus, and medial frontal regions consistent with the additional demands of perspective taking and social cognition.^11^ The form of inner speech engaged during experimental tasks may also differ from spontaneously occurring inner speech; in a combined functional magnetic resonance imaging (fMRI) and experience-sampling study, Hurlburt et al. (2016)^12^ found that task-elicited and spontaneous inner speech produced opposing patterns of activation in Heschl’s gyrus and differing effects in left inferior frontal gyrus, raising the possibility that the generalization of results to everyday inner speech by laboratory paradigms is not straightforward.

Despite this growing recognition of the cortical complexity of inner speech, existing work shares a common limitation: it has focused almost exclusively on the cortical level, treating speech-production networks as if they operated in isolation from the systems that regulate their functional state. Cortical circuits, however, do not function autonomously; their moment-to-moment processing is shaped by ascending neuromodulatory systems that set the state in which cortical computations unfold. Recent mouse and human neuroimaging work indicates that pupil-indexed arousal provides a compact readout of large-scale brain dynamics, predicting much of cortex-wide spatiotemporal physiology in mice^13^ and indexing whole-brain integration states in humans.^14^ These recent findings point to arousal regulation as a key to the higher-order cortical activity and the spontaneous whole-brain fluctuations in which human inner speech is itself embedded. This raises a question that the cortical-overlap framework, however refined, cannot answer: even when covert and overt speech recruit similar cortical regions, are these regions in the same functional state during covert and overt speech?

Indeed, the dependence of cortical processing on neuromodulatory state is well documented. For instance, the locus coeruleus-noradrenergic system exerts an adaptive-gain function that adjusts cortical responsivity and tunes the signal-to-noise ratio of ongoing processing.^15^ The lateral prefrontal cortex depends on monoaminergic and cholinergic neuromodulation for its proper function,^16^ and these systems actively shape how cortical circuits process information—as shown in the auditory cortex, where arousal state determines the quality and reliability of sound-evoked neural responses.^17^^,^^18^ A comparable dependence holds for language-related networks, where the disruption of the neuromodulatory input is associated with impaired language processing and poor recovery from aphasia.^19^ We, therefore, speculate that arousal systems may underlie any cortical similarity or dissimilarity between the two speech modalities. Yet, whether inner and overt speech differentially engage arousal systems has not been directly examined.

Pupillometry provides a non-invasive window into the arousal systems described above. Recent studies have begun to reveal the detailed mechanisms underlying pupil-linked arousal, and among autonomic indices, the pupil is believed to closely track brain functional states,^13^^,^^20^^,^^21^ a notable feature that other brain-imaging methods lack.^22^^,^^23^ Pupil-linked arousal tracks rapid changes in the cortical and neuromodulatory states^17^^,^^18^ and covaries with the activity in several ascending neuromodulatory systems that regulate arousal, including the noradrenergic locus coeruleus and cholinergic basal forebrain.^20^^,^^24^ It also predicts aspects of subcortical and cortical arousal.^13^^,^^20^^,^^21^^,^^24^^,^^25^^,^^26^^,^^27^ It is also subject to top-down executive control^28^ and reliably increases in response to cognitive effort, respiration, and physical activity.^26^^,^^29^^,^^30^^,^^31^^,^^32^^,^^33^ Such state-dependent modulation may also extend to the auditory and language-related cortical systems on which speech production and perception depend, raising the possibility that pupil-linked arousal could be associated with the functional state in which overt and inner speech are processed in humans. Consistent with this possibility, in audiological research, the task-evoked pupil dilation response has been widely used as an index of listening effort, increasing with auditory task difficulty such as lower speech intelligibility or signal-to-noise ratio.^34^^,^^35^ Interestingly, in animal research, pupil-linked arousal has been shown to track the cortical state and modulate the membrane-potential dynamics, neural excitability, variability, tuning, and sensory encoding, including in the auditory cortex.^17^^,^^18^^,^^36^^,^^37^^,^^38^ Movement-related active states, often accompanied by pupil dilation, can also suppress or diversely reshape sound-evoked responses in the auditory cortex.^24^ From a methodological standpoint, pupillometry can be performed continuously and non-invasively, requires no head-mounted devices, and remains robust during overt articulatory movements, making it uniquely suited for the direct comparison of inner and overt speech within a single paradigm. In contrast, other neuroimaging methods are often susceptible to significant speech-related noise,^1^^,^^39^ and their head- or face-mounted apparatus may compromise the more natural, ecologically valid speech that such comparisons require. Although pupillary responses during speech production have not yet been well studied, it has been suggested that overt vocalization elicits pupillary dilation.^40^^,^^41^ Inner speech is known to recruit cortical processes that partly overlap with overt speech, including left-lateralized language regions.^1^^,^^2^ Moreover, inner speech may place demands on sustained attention and verbal working memory, particularly when phonological representations must be internally maintained and monitored.^1^ Some accounts further suggest that inner speech may engage motor-inhibitory control to prevent overt articulation.^6^^,^^42^ Additionally, motor imagery tasks have been shown to elicit pupil dilation,^43^^,^^44^ suggesting that pupil-linked arousal is engaged even when overt action is suppressed. Importantly, pupillometry does not provide a direct measure of inner speech itself; rather, it provides a non-invasive physiological index of the arousal-related processes that may accompany covert verbal processing. On this basis, we hypothesized that pupil-linked arousal would also be engaged during inner speech, reflecting the cortical and cognitive demands it recruits.

In the present study, we addressed this question using a numerical counting paradigm (Figure 1). Counting provides a useful experimental model because it is a highly controlled verbal task with minimal affective variability, can be implemented in both inner and overt forms, and allows systematic manipulation of cognitive load while preserving the core structure of verbal production. It also makes it relatively feasible to experimentally verify, to some extent, whether inner speech is being engaged. Our recent work, for example, used numerical counting to show that preschool children (3–6 years old) are capable of inner speech to a measurable degree.^45^ Although this paradigm does not capture the full diversity of naturally occurring inner speech, it provides a controlled model of one common and experimentally tractable form: silent verbal counting. This form resembles everyday internal speech, such as silently counting objects and covert verbal rehearsal,^46^ but it is also externally instructed, repetitive, and relatively limited in the semantic, affective, and social content. Moreover, task-elicited inner speech may differ neurologically from spontaneous inner speech in daily life.^12^ Thus, the present findings should be interpreted as evidence concerning task-elicited inner counting, rather than inner speech as a whole. Despite these constraints, the paradigm was adopted because it allowed inner and overt speech to be compared under closely matched verbal and temporal conditions, while cognitive load was manipulated systematically. We, therefore, first tested whether inner speech engages pupil-linked arousal in a manner comparable to overt speech, using a simple numerical counting paradigm (experiment 1). Contrary to our primary hypothesis, inner speech produced, not dilation, but constriction relative to baseline, while overt speech led to dilation as expected, raising the question of whether this dissociation is a robust feature of covert verbal production or specific to the demands of simple numerical counting. We next examined whether the dissociation persists when verbal content is enriched; in experiment 2, participants counted using grammatically structured numeral-noun phrases, a manipulation that increases phonological and morphological complexity, while preserving the counting structure. Finally, in experiment 3, we asked whether the dissociation holds under a higher cognitive load by requiring step counting, thereby examining whether inner speech arousal scales with task demands. This stepwise approach allowed us to characterize the conditions under which pupil-linked arousal during inner speech is lower than during overt speech and the extent to which that difference varies with the verbal content and cognitive demands.

**Figure 1:**
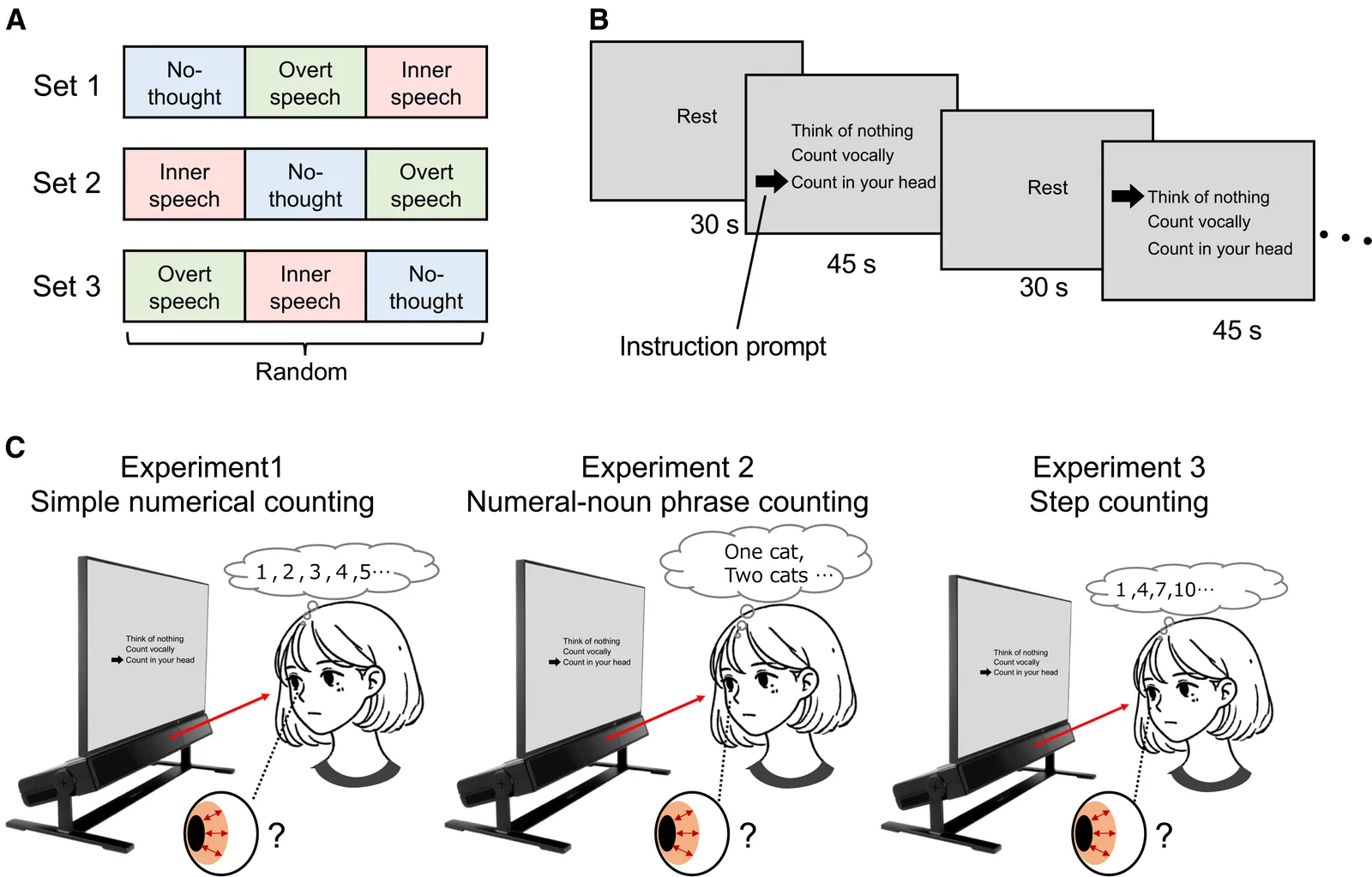
Schematic of the experimental paradigm and counting tasks (A) Experimental session structure. Each participant completed three sets. Each set comprised one block of each of the three conditions: no-thought (a control condition requiring participants to count neither aloud nor silently and to refrain from deliberate thought), overt speech (counting aloud), and inner speech (counting silently, in the head). The order of the conditions was randomized within each set. (B) Example trial sequence. Each block consisted of a 30-s rest period followed by a 45-s task period. During task periods, participants were prompted with an arrow indicating which instruction to follow: no-thought, overt speech, and inner speech. (Note: actual on-screen instructions were presented in Japanese: “休み” for rest, “何も考えない” for “no thought,” “声に出して数える” for overt speech, and “心の中で数える” for inner speech). (C) Experimental setup and task overview. Participants maintained their gaze on an on-screen instruction panel, while pupil diameter was continuously recorded using a screen-based eye tracker. The illustrations depict the inner-speech condition across the three experiments. In experiment 1, the participants counted upward from 1 using consecutive integers. In experiment 2, the participants counted in Japanese using grammatically structured numeral-classifier phrases (e.g., “one cat, two cats … ”). In experiment 3, the participants performed step counting in increments of 2, 3, or 4 (an increment of 3 is illustrated here).

## Results

### Validation of inner speech compliance

As an indirect check of compliance, we examined the correlation between the final numbers reported in the overt- and inner-speech conditions. Participants who counted relatively quickly during overt speech were expected to also do so during inner speech if they were engaging in the instructed covert counting process. Consistent with this, strong positive correlations were observed in experiment 1 (rho = 0.92, *p* < 0.001), experiment 2 (rho = 0.85, *p* < 0.001), and experiment 3 (rho = 0.89, *p* < 0.001) (Figure 2). Mean final counts (inner speech vs. overt speech) were 46.9 vs. 45.2 in experiment 1 (*t*(22) = 0.70, *p* = 0.491, Cohen's *d*z = 0.15), 33.6 vs. 29.7 in experiment 2 (*t*(26) = 4.10, *p* < 0.001, *d*z = 0.79), and 100.4 vs. 101.7 in experiment 3 (averaged across step sizes of 2, 3, and 4) (*t*(29) = 0.56, *p* = 0.578, *d*z = 0.10). Together, despite the absolute count difference in experiment 2, these highly correlated findings provide indirect support for compliance with the inner-speech instructions. In supporting analyses, self-report ratings of task-unrelated thought during the no-thought condition remained low across experiments, and the absolute baseline pupil diameter did not differ among the three experiments (Data S1 and Figure S1).

**Figure 2:**
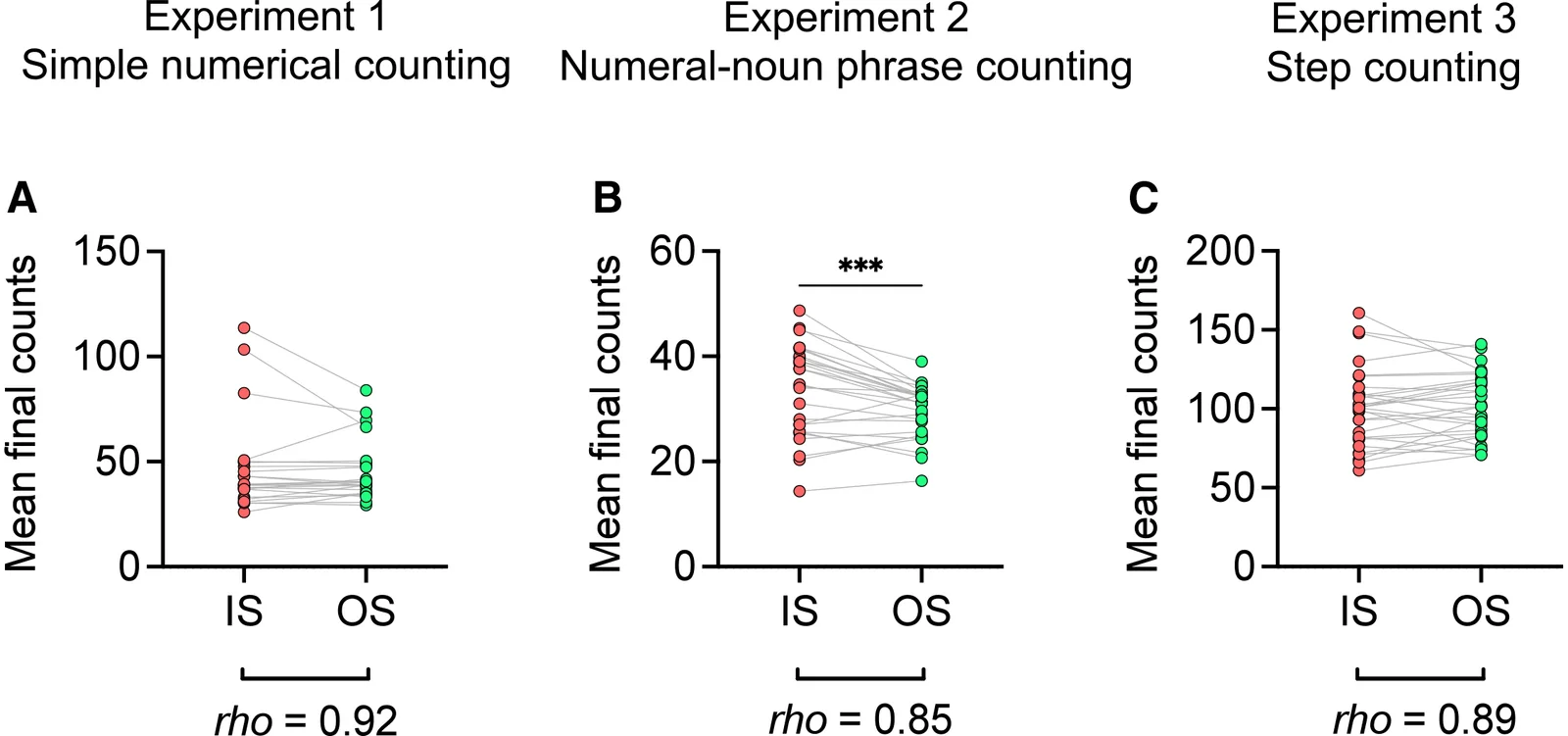
Final counts in the inner- and overt-speech conditions as a compliance check (A–C) Mean final counts for inner speech (IS) and overt speech (OS) in (A) experiment 1 (simple numerical counting), (B) experiment 2 (numeral-noun phrase counting), and (C) experiment 3 (step counting); each line connects an individual participant. Spearman’s correlations between conditions were strong in every experiment (rho = 0.92, 0.85, and 0.89 for experiments 1, 2, and 3, respectively). In experiment 2, final counts were higher during IS than during OS (∗∗∗*p* < 0.001).

### Pupillometry

#### Experiment 1: Simple numerical counting

Baseline-adjusted pupil diameter differed significantly across conditions (repeated-measures one-way analysis of variance [ANOVA]: *F*(1.50, 33.10) = 9.05, *p* = 0.002, partial *η*^*2*^ = 0.29) (Figures 3A and 3B). Contrary to our initial hypothesis, pupillary responses during inner speech were significantly smaller than those during no-thought (*t*(22) = 3.20, adjusted *p* = 0.012, *d*z = 0.67) and overt speech (*t*(22) = 3.55, adjusted *p* = 0.005, *d*z = 0.74), whereas no significant difference was observed between no-thought and overt speech (*t*(22) = 1.99, adjusted *p* = 0.178, *d*z = 0.41). To clarify the direction of these effects relative to baseline, one-sample tests showed that overt speech elicited significant pupil dilation (*t*(22) = 2.22, *p* = 0.038, *d*z = 0.46), whereas inner speech elicited significant pupil constriction (*t*(22) = −2.89, *p* = 0.009, *d*z = −0.60). The no-thought condition did not differ from baseline (*t*(22) = 0.65, *p* = 0.524, *d*z = 0.13).

**Figure 3:**
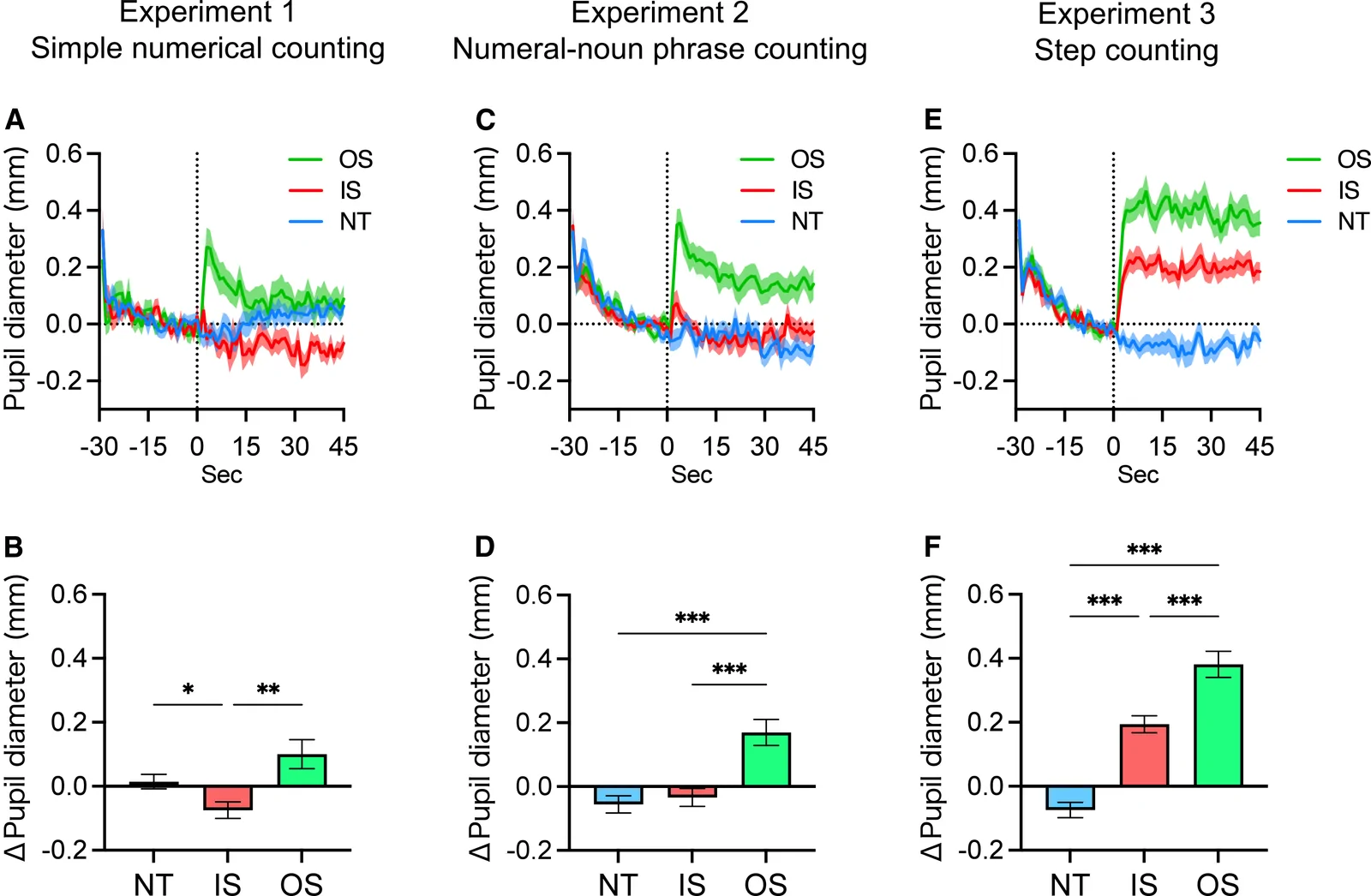
Pupil change between inner and overt speech across experiments (A, C, and E) Time courses of baseline-adjusted pupil diameter across the 30-s rest and 45-s task periods. The vertical dotted line at 0 s indicates the onset of the task. Solid lines represent the mean pupil diameter for the overt-speech (OS, green), inner-speech (IS, red), and no-thought (NT, blue) conditions. Shaded areas indicate the standard error of the mean (SEM). Data represent experiment 1 (simple numerical counting) (A), experiment 2 (numeral-noun phrase counting) (C), and experiment 3 (step counting) (E). (B, D, and F) Mean baseline (15 s pre-task)-adjusted pupil change (Δ pupil diameter) during the 45-s task period for experiment 1 (B), experiment 2 (D), and experiment 3 (F). Error bars represent SEM. Asterisks indicate significant differences between conditions assessed via post hoc paired-samples *t* tests following a significant repeated-measures ANOVA (∗*p* < 0.05, ∗∗*p* < 0.01, ∗∗∗*p* < 0.001; *n* = 23, 27, and 30 participants in experiments 1, 2, and 3, respectively).

#### Experiment 2: Numeral-noun phrase counting

Baseline-adjusted pupil diameter differed significantly across conditions (repeated-measures one-way ANOVA: *F*(1.48, 38.53) = 18.91, *p* < 0.001, partial *η*^*2*^ = 0.42) (Figures 3C and 3D). Consistent with experiment 1, pupil size during overt speech was significantly larger than those during no-thought (*t*(26) = 4.45, adjusted *p* < 0.001, *d*z = 0.86) and inner speech (*t*(26) = 5.41, adjusted *p* < 0.001, *d*z = 1.04). However, contrary to our pre-registered hypothesis (https://osf.io/7efbv) based on experiment 1, inner speech and no-thought did not differ significantly (*t*(26) = 0.71, adjusted *p* > 0.999, *d*z = 0.14). To clarify the direction of these effects relative to baseline, we used one-sample t tests, which showed that overt speech elicited significant pupil dilation (*t*(26) = 4.13, *p* < 0.001, *d*z = 0.80), whereas no-thought showed a small but significant pupil constriction (*t*(26) = −2.06, *p* = 0.049, *d*z = −0.40). Inner speech did not significantly differ from baseline (*t*(26) = −1.22, *p* = 0.234, *d*z = −0.23).

#### Experiment 3: Step counting

Baseline-adjusted pupil diameter differed significantly across conditions (repeated-measures one-way ANOVA: *F*(1.53, 44.42) = 56.67, *p* < 0.001, partial *η*^*2*^ = 0.66) (Figures 3E and 3F). During counting with step sizes of 2, 3, and 4, pupillary responses differed across all three conditions: inner speech responses were significantly larger than those during no-thought (*t*(29) = 6.75, adjusted *p* < 0.001, *d*z = 1.23) but significantly smaller than those during overt speech (*t*(29) = 5.57, adjusted *p* < 0.001, *d*z = 1.02), and overt speech also elicited significantly larger responses than no-thought (*t*(29) = 8.55, adjusted *p* < 0.001, *d*z = 1.56). To clarify the direction of these effects relative to baseline, one-sample tests showed that no-thought elicited a small but significant pupil constriction (*t*(29) = −3.13, *p* = 0.004, *d*z = −0.57), whereas both inner speech (*t*(29) = 7.31, *p* < 0.001, *d*z = 1.34) and overt speech (*t*(29) = 9.33, *p* < 0.001, *d*z = 1.70) elicited significant pupil dilation. Effects of step size are reported in Figure S2.

## Discussion

The results of the present study do not support the initial assumption that inner and overt speech produce equivalent pupillary responses. Across the three experiments, overt speech consistently elicited pupil dilation, whereas inner speech did not. Critically, the direction of the inner-speech pupillary response relative to baseline was not fixed: pupil constriction occurred during simple numerical counting (experiment 1), no significant deviation from baseline occurred during noun-phrase counting (experiment 2), and clear dilation occurred during cognitively demanding step counting (experiment 3). Together, these findings indicate that inner speech was consistently associated with lower pupil-linked arousal than overt speech in every experiment, even though the direction of the inner-speech response relative to baseline varied systematically with the verbal content and cognitive demands of the task.

### Task validity and control conditions

A distinctive strength of the present study is that inner speech, overt speech, and an instructed no-thought condition were compared within a single, closely matched counting paradigm. Two features support this interpretation. First, counting speed was strongly correlated between overt and inner speech (rho > 0.85 across all three experiments), indicating that the participants genuinely performed inner counting. Only in experiment 2 did the participants count to higher numbers during inner speech within the same time window, indicating faster progression, consistent with reports that inner speech can proceed faster than overt speech.^47^

Second, although the no-thought condition was an instructed control rather than a neutral physiological zero point, our main conclusions rest on the robust within-experiment dissociation between inner and overt speech. This relative control condition, nonetheless, provided a valuable within-experiment reference, anchoring the divergent pupil responses of inner and overt speech. The participants’ post-task ratings indicated that they generally sustained the no-thought state, although these self-reports cannot verify complete mental silence (Data S1).

### Overt and inner speech show distinguishable pupil-linked arousal profiles

The most robust finding across all three experiments was the consistent pupil dilation elicited by overt speech, suggesting that the presence of vocalization is an important factor shaping pupil-linked arousal. Pupil diameter is a well-established index of cognitive effort and brain arousal state,^25^^,^^29^ reflecting coordinated activity across the locus coeruleus-noradrenergic system,^27^^,^^48^ the basal forebrain cholinergic system,^24^ the orexin system,^49^ and associated cortical network states.^13^^,^^21^ In recent work, pupil-linked arousal has further been associated with brain-wide neural dynamics.^13^ Although pupillometry has been applied to memory load,^29^ lexical processing,^50^ speech planning,^50^ vocal reading,^40^^,^^41^ musical imagery,^51^ and physical activity,^31^ direct comparisons of inner speech, overt speech, and instructed no-thought within a matched task structure are, to our knowledge, absent. This dilation likely reflects the integrated pupil-linked arousal recruitment associated with motor execution, respiratory variation, sensorimotor feedback, and attention,^27^^,^^30^^,^^41^^,^^52^ each of which is presumed to engage brainstem arousal systems and cortical network states,^21^^,^^52^ potentially contributing to efficient speech production.^16^^,^^19^ Inner speech, by contrast, did not approach overt-speech levels of pupil dilation in any condition, suggesting that covert verbal production can be executed without recruiting pupil-linked arousal systems and cortical networks to the same degree.

Interestingly, inner speech was not uniformly associated with low pupil-linked arousal. Under a minimal cognitive load (experiment 1), inner speech produced pupil constriction, even relative to the no-thought condition, suggesting that highly routinized covert counting is accompanied by a reduced pupil-linked arousal response. As linguistic complexity increased (experiment 2), this constriction was abolished, and inner speech no longer differed from baseline. Under the highest load (experiment 3), inner speech produced clear dilation, converging toward—but remaining significantly below—that of overt speech. Inner speech, therefore, maintains consistently lower pupil-linked arousal than overt speech while tracking cognitive load in a qualitatively similar manner. This indicates that pupil-linked arousal during inner speech scales with cognitive-control demand rather than with linguistic complexity per se: in experiment 2, greater linguistic and phonological complexity alone did not raise arousal, whereas the higher updating and control demands of experiment 3 did. That patterns across experiments differed relative to baseline, therefore, does not undermine the main interpretation but, rather, specifies it, with overt speech reliably recruiting pupil-linked arousal and inner speech showing a task-dependent profile rather than a lower-amplitude copy of overt speech.

### Toward a state-dependent neurophysiological account of inner speech

The varying direction of the inner-speech pupil response across experiments 1–3 (constriction, no reliable change, and limited dilation) also connects to the broader neuroscience of inner speech. Past neuroimaging studies have established that inner and overt speech partially share language-related networks and speech production processes,^4^^,^^5^ and inner speech has been discussed as a form of inhibited overt speech.^42^ Our findings do not challenge this shared substrate; they instead reveal a distinguishable pupil-linked arousal profile for inner speech that need not imply reduced cognitive engagement. However, the picture is more complex: the neuroimaging literature has not converged on a single account of how covert and overt speech relate at the cortical level, with inner speech showing weaker, comparable, or occasionally greater activation than overt speech depending on the region and task.^1^^,^^3^^,^^6^^,^^7^ Against this heterogeneous background, such variability is unsurprising in light of the present findings. The one consistent pattern was that, in every experiment, inner speech was accompanied by lower pupil-linked arousal than overt speech. We propose that inner and overt speech differ not only in which cortical regions they engage but also in the overall pupil-linked arousal state in which those regions operate. Because pupil-linked arousal is thought to track coordinated, brain-wide fluctuations in neural activity,^13^ a shift in this state may modulate how those shared networks function. This may be one reason why imaging studies report mixed cortical differences between inner and overt speech, rather than consistent evidence that the two rely on entirely separate circuits. This view also makes a testable prediction: in more varied, naturalistic inner speech, pupil-linked arousal should rise and fall with task difficulty, so that its cortical and pupillary signatures shift together with cognitive demand.

This state-dependent view also helps clarify why inner speech did not exhibit a fixed pupil response across experiments. Inner speech is not a unitary phenomenon: it can remain relatively condensed, with sufficient semantic and contextual information and full phonological-articulatory specification being unnecessary, or it can become more expansive when the task requires active verbal maintenance, updating, or rehearsal.^4^ More tentatively, the pupil-linked arousal reflected by the clear increase in dilation observed between experiment 2 and experiment 3 may support a more expanded form of inner speech (typically engaged under stress or high cognitive demand^4^), thereby aiding the verbal maintenance and updating it requires, a contribution largely absent in experiments 1 and 2.

This interpretation also provides a possible mechanistic account of the robust overt-speech effect. Overt speech requires an internal linguistic representation to be transformed into an externally executable sensorimotor format, including phonological specification, articulatory motor execution, respiratory coordination, auditory feedback monitoring, and comparison with an efference copy of the motor command.^9^^,^^53^ Consistent with this view, animal auditory-cortical studies have shown that pupil-linked arousal and movement-related active states modulate membrane-potential dynamics, excitability, spontaneous and sound-evoked firing, sensory gain, and stimulus coding, with movement often suppressing or diversely reshaping sound-evoked responses.^17^^,^^18^^,^^36^^,^^37^ Just as movement reshapes the cortical state in the animal models above, the motor and respiratory demands of overt speech may engage comparable arousal-related modulation of auditory–motor cortex in humans. These additional demands may recruit stronger arousal-related gain control than inner speech, which can often remain in a compressed representational format. A natural candidate mechanism is the locus coeruleus-noradrenergic system, whose adaptive-gain function adjusts cortical responsivity and tunes the signal-to-noise ratio of ongoing processing.^15^^,^^16^ The greater pupil-linked arousal observed during overt speech may, therefore, reflect stronger noradrenergic gain modulation of speech-related auditory-motor networks, whereas inner speech may engage similar linguistic machinery at a lower gain. On this account, the cortical overlap between inner and overt speech reflects shared linguistic machinery, whereas their dissociable pupil-linked arousal might reflect the different sensorimotor and arousal states in which that machinery is run. Although mouse auditory-cortical mechanisms and human overt and inner speech are separated by a substantial translational gap, pupil-linked arousal provides a useful bridge between animal neurophysiology and human speech cognition, and the present study offers an initial step in this direction.

### Implications and significance

Given that pupillometry is non-invasive and contactless, these findings open avenues for examining how pupil-linked arousal changes as overt speech becomes internalized across developmental phases and individuals. If this pattern extends beyond task-elicited inner counting, the capacity of inner speech to sustain verbal processing with reduced pupil-linked arousal may be advantageous where efficient cognitive activity and mental calm must coexist—as in cognitive control,^54^ sport performance,^54^ or routinized inner counting as a sleep aid^55^—yet may also enable maladaptive cognitive loops that contribute to rumination.^56^ Conversely, the elevated pupil-linked arousal accompanying overt speech may carry functional value; Fawcett et al.^40^ found that pupil dilation that occurs while reading aloud better tracks the memory advantage for spoken words than for silent words.

The magnitude of the overt-speech response is itself notable: the sustained dilation in experiment 3, approximately 0.4 mm for more than 10 s, is large relative to those reported in prior pupillometry studies and comparable in magnitude to responses reported during high-intensity cycling at roughly 80% of maximal oxygen uptake.^23^^,^^31^ This places overt speech under cognitive load on the scale of vigorous physical exertion, underscoring how much arousal vocalization can add. Because moderate arousal is thought to support optimal function (the Yerkes-Dodson law),^17^ inner speech might also limit the excessive arousal that arises when cognitive load and vocalization coincide, consistent with reviews noting that self-talk is widely used in sport to regulate arousal.^54^

To our knowledge, the present work represents the introduction of pupil-linked arousal and its underlying neuromodulatory systems as a source of analysis complementary to the cortical focus that has dominated inner speech research. Because the present results reveal dissociable pupil-linked arousal during overt and inner speech, the approach can contribute to questions and theory building across domains: how verbal thought is generated and controlled, how silent self-talk supports self-regulation and self-awareness, how inner speech becomes internalized over development, and what changes when it is misattributed, as in auditory verbal hallucinations? Methodologically, pupillometry enables a continuous, closely matched comparison of covert and overt speech that circumvents the articulatory, orofacial, respiratory, and movement artifacts that confound electroencephalography (EEG), magnetoencephalography (MEG), functional near-infrared spectroscopy (fNIRS), and facial electromyography (EMG) during overt articulation. Moreover, being non-invasive, contact-free, and silent, it may be suitable for future studies in populations for whom overt report or sensor-based recording is difficult, such as young children and certain clinical groups. We, therefore, view this work as a focused first step that, across three experiments including a replication and a cognitive-load manipulation, provides convergent evidence for a consistent attenuation of pupil-linked arousal during inner relative to overt speech.

In summary, across three experiments, overt speech consistently induced pupil dilation, whereas inner speech was accompanied by lower pupil-linked arousal, with a baseline-relative response that varied with task demands from constriction to no reliable change to dilation under high load. Within the present task-elicited counting paradigm, inner and overt speech were, therefore, associated with dissociable pupil-linked arousal, indicating that inner speech is not simply a lower-amplitude, silent version of overt speech.

### Limitations of the study

First, the present study operationalized inner speech as silent numerical counting. This paradigm allowed us to compare inner and overt speech under closely matched task conditions and to manipulate cognitive load systematically. However, it provides only a constrained model of inner speech. Numerical counting is externally instructed, repetitive, and relatively limited in the semantic, affective, and social content. It, therefore, cannot capture the full diversity of naturally occurring inner speech.^11^^,^^12^ Accordingly, the present findings should be interpreted as evidence for pupil-linked arousal during task-elicited inner counting, rather than as a general account of inner speech as a whole. Future studies should examine whether similar pupil-linked arousal profiles are observed during more naturalistic forms of inner speech, such as self-generated verbal thought, autobiographical reflection, dialogic inner speech, or experience-sampling paradigms. Second, the no-thought condition reflected an instructed attempt to refrain from deliberate thought rather than verified mental silence. Mind wandering cannot be entirely eliminated, and this condition should, therefore, be regarded as an imperfect, instructed control condition, rather than a verified state of mental silence.^4^ Third, we relied on pupillometry as our sole and, necessarily indirect, physiological index of arousal. Although the present results further highlight the promise of pupil diameter as a tool for examining how inner and overt speech differ, they do not imply that changes in pupil size alone can provide a readout of inner speech itself. Promising next steps include further analyses of pupil oscillatory dynamics and multimodal approaches that combine pupillometry with converging measures such as fMRI, EEG, heart rate variability, electrodermal activity, respiration, and self-reported effort. Fourth, the sex ratio was unbalanced and differed across the experiments (experiment 1: 14 females/9 males; experiment 2: 8 females/19 males; experiment 3: 14 females/16 males), and it also warrants caution when comparing the experimental results.

## Resource availability

### Lead contact

Requests for further information and resources should be directed to and will be fulfilled by the lead contact, Ryuta Kuwamizu (kuwamizu.ryuta.ft@u.tsukuba.ac.jp).

### Materials availability

This study did not generate new unique reagents or materials.

### Data and code availability

• Data: Data have been deposited at Figshare and are publicly available at https://doi.org/10.6084/m9.figshare.33794563.
• Code: Code has been deposited at Figshare and is publicly available at https://doi.org/10.6084/m9.figshare.33794563.
• All other items: Additional information is available from the lead contact upon request.

## Acknowledgments

The authors express their gratitude to Ms. Noguchi (ELCS English Language Consultation Services, Japan) for helping with the manuscript. They thank R. Hosokawa (Tokyo Institute of Technology, Japan) for analysis support. R.K. was supported by 10.13039/501100001691JSPS 10.13039/501100001691KAKENHI (grant numbers 23H04830, 24K20598, and 23KJ1169). Y.M. was supported by 10.13039/501100001691JSPS 10.13039/501100001691KAKENHI (grant numbers 24K00486 and 23H04832). R.W., N.Y., and R.K. were supported by a grant from Advanced Research Initiative for Human High Performance (ARIHHP), 10.13039/501100006559University of Tsukuba. K.O., R.W., N.Y., and R.K. also acknowledge the financial support from a crowdfunding project through the academic crowdfunding platform “academist” (project no. 396).

## Author contributions

Conceptualization, R.K., R.W., K.O., and Y.M.; methodology, R.K. and R.W.; experiment 1, R.K. and K.O.; experiments 2 & 3, R.W.; participant recruitment, N.Y.; data analysis, R.K.; visualization, R.K.; writing – original draft, R.K.; writing – review & editing, R.W., N.Y., K.O., and Y.M.; project administration, R.K.; funding acquisition, R.K., R.W., N.Y., K.O., and Y.M.; supervision, Y.M.

## Declaration of interests

The authors declare no competing interests.

## Declaration of generative AI and AI-assisted technologies in the manuscript preparation process

During the preparation of this manuscript, the authors used Anthropic Claude (Opus 4.6) for checking English wording and expression. The authors carefully reviewed, edited, and rewrote all AI-assisted output, and the manuscript was subsequently checked by a native English-speaking proofreader. The authors take full responsibility for the content. AI was not used to create figures.

## STAR★Methods

### Key resources table

**Table:** 

REAGENT or RESOURCE	SOURCE	IDENTIFIER
**Deposited data**		

Raw and processed pupillometry and behavioral data, and GraphPad Prism files used for statistical analysis and figure generation	This paper	figshare: https://doi.org/10.6084/m9.figshare.33794563

**Software and algorithms**		

Tobii Pro Lab (stimulus presentation and pupil data acquisition/processing)	Tobii AB, Danderyd, Sweden; https://www.tobii.com/products/software/behavior-research-software/tobii-pro-lab	Version 25.7.1400 (x64); RRID not available
GraphPad Prism 9 (macOS; statistical analysis and figure generation)	GraphPad Software, San Diego, CA, USA; https://www.graphpad.com/	Version 9.5.1 (528); RRID:SCR_002798
G∗Power (*a priori* power analysis)	Faul et al., 2007^57^; https://www.psychologie.hhu.de/arbeitsgruppen/allgemeine-psychologie-und-arbeitspsychologie/gpower	Version 3.1; RRID:SCR_013726
Original code (Python; pupil binning)	This paper	figshare: https://doi.org/10.6084/m9.figshare.33794563
Python	Python Software Foundation; https://www.python.org/	RRID:SCR_008394

**Other**		

Tobii Pro Spectrum eye tracker (300 Hz model; data acquired at 60 Hz)	Tobii AB, Danderyd, Sweden; https://www.tobii.com/products/eye-trackers/screen-based/tobii-pro-spectrum	N/A (catalog number not available)
FlexScan EV2451 monitor (23.8 in)	EIZO Corporation, Japan; https://www.eizo.co.jp/support/db/products/model/EV2451	Cat# EV2451
Digital Lux Meter, model 78747	Shinwa Rules Co., Ltd., Japan; https://www.shinwasokutei.co.jp/	Model 78747

### Experimental model and study participant details

Across all three experiments, participants were healthy, native Japanese-speaking undergraduate and graduate students with normal or corrected-to-normal vision; glasses or contact lenses were permitted and recorded. The study was conducted in accordance with the Declaration of Helsinki and was approved by the Ethics Committee of the Unit for Advanced Studies of the Human Mind, Kyoto University (28-P-12). All participants provided written informed consent prior to participation.

In Experiment 1, 25 individuals were recruited, yielding a final sample of 23 (mean age = 21.65 years, SD = 3.02; 14 females, 9 males) after excluding 2 participants due to excessive missing pupil data (see *Quantification and statistical analysis*). In Experiment 2, 30 individuals were recruited, yielding a final sample of 27 (mean age = 20.23 years, SD = 2.05; 8 females, 19 males) after excluding 3 participants for the same reason. In Experiment 3, 30 individuals were recruited with no exclusions, yielding a final sample of 30 (mean age = 21.97 years, SD = 6.37; 14 females, 16 males).

Participants requiring no vision correction, glasses, and contact lenses numbered 12, 4, and 7 in Experiment 1; 9, 9, and 9 in Experiment 2; and 7, 10, and 13 in Experiment 3, respectively. Participants reporting caffeine intake on the day of testing numbered 8, 5, and 11 across Experiments 1–3, respectively.

### Method details

#### Apparatus and stimulus presentation

Stimuli were presented, and pupil diameter was acquired and processed, using Tobii Pro Lab (Tobii AB, Danderyd, Sweden) with a screen-based eye tracker (Tobii Pro Spectrum, Tobii AB; a 300 Hz-capable model operated at a 60 Hz acquisition rate). Visual stimuli were shown on a 23.8-inch (60 cm) monitor (FlexScan EV2451, EIZO, Japan). A 9-point calibration was performed at the beginning of each session. Participants were seated approximately 70 cm from the display and were instructed to maintain fixation and minimize head movements.

To minimize luminance-related confounds, visual parameters were strictly matched across the three conditions. All screens used a uniform gray background (RGB: 120, 120, 120), and all task-relevant symbols and letters were rendered in black (RGB: 0, 0, 0).^23^^,^^27^^,^^31^ On-screen instructions were presented in Japanese using the Meiryo UI font at size 48. The three conditions differed only in the on-screen position of the instruction arrow, ensuring that luminance and the amount of on-screen visual information remained constant across conditions. Illuminance in the testing room was monitored with a digital lux meter (model 78747; Shinwa Rules Co., Ltd., Japan); the light sensor was placed on the experimental desk facing upward, and illuminance was maintained at 240–340 lux throughout.

#### Experimental design and procedure

Across all experiments, participants completed a block-designed task while maintaining their gaze on an on-screen instruction panel. Each block consisted of a 30-s rest period followed by a 45-s task period. During the rest period, the word “Rest” was displayed; the first 15 s served as a washout interval and the last 15 s defined the baseline period for pupillometry. During both rest and task periods, participants kept looking at the screen and minimized head movements.

During task periods, participants were prompted with an arrow indicating which of three instructions to follow: (1) No-thought (attempt to think of nothing), (2) Overt speech (count aloud), and (3) Inner speech (count silently in the mind). One set comprised one block of each condition, and each participant completed three sets (three blocks per condition). The order of conditions was randomized. Pupil diameter was recorded continuously. No instruction was given regarding counting pace; participants counted at their own natural tempo. Compliance was assessed by asking participants to report the final number reached during the overt- and inner-speech conditions. On-screen instructions were presented in Japanese (“休み” for Rest, “何も考えない” for No-thought, “声に出して数える” for Overt speech, and “心の中で数える” for Inner speech).

#### Experiment 1: Simple numerical counting

Participants counted upward from 1 using consecutive integers (e.g., 1, 2, 3, 4 …). Compliance questions were administered after each session.

#### Experiment 2: Numeral-noun phrase counting

Participants counted in Japanese using grammatically structured numeral–classifier phrases that included the nominative particle *ga* (が) and category-specific classifiers. Before each session, the experimenter specified the semantic category (cats, balls, or books), and participants generated sequential counting phrases accordingly (e.g., *neko ga ippiki, neko ga nihiki, neko ga sanbiki* … for the animal category). This manipulation introduced semantic variation and Japanese-specific morpho-phonological alternations across numbers (e.g., gemination and voicing), thereby increasing phonological/linguistic variability relative to simple numeral counting. The order of semantic categories was randomized and counterbalanced across participants. Compliance questions were administered after each block, and the next rest period began once the participant had responded.

#### Experiment 3: Step counting

Experiment 3 was designed to test whether the inner–overt speech dissociation generalizes to a more cognitively demanding task while preserving the verbal-counting structure of Experiments 1 and 2. Step counting was chosen because it retains this counting structure while increasing demands on working memory, updating, and cognitive control. Participants performed step counting. Before each session, the experimenter explained the rule: counting upward from 1 in increments of 2, 3, or 4 (e.g., 1, 3, 5, 7 …; 1, 4, 7, 10 …; 1, 5, 9, 13 …). The order of increment size was randomized and counterbalanced across participants. Compliance questions were administered in the same manner as in Experiment 2.

#### Pupil recording and preprocessing

Pupil diameter was recorded continuously at 60 Hz and processed in Tobii Pro Lab. Following previous work,^31^,^32^^,^^58^^,^^59^ only valid samples from the right eye were analyzed. Missing or invalid samples (e.g., due to blinks or gaze loss) were excluded automatically. Baseline-adjusted pupil change (Δpupil diameter, mm) was computed as the mean pupil diameter during the 45-s task period minus the mean baseline value (the last 15 s of the preceding rest period). Pupil data were downsampled to 1 Hz by taking the median within each 1-s bin, reducing the influence of transient spikes and brief artifacts, and the 1-Hz time series was then averaged within each trial.

#### Compliance and no-thought rating

As an indirect compliance check for inner speech, the final numbers reported in the overt- and inner-speech conditions were compared within participants (see Quantification and statistical analysis). In addition, a single 0–10 self-report rating (0 = thought about nothing at all; 10 = thought about various things) of the degree to which participants had been thinking about something during the no-thought condition was collected (Experiment 1, after the session [available for 20 of 23 participants]; Experiments 2 and 3, after each block); this rating and its results are reported in Data S1.

### Quantification and statistical analysis

#### Sample size determination

Sample sizes were determined *a priori* in G∗Power. The required sample size was computed for both the omnibus repeated-measures ANOVA and the pairwise post hoc contrasts; because the pairwise contrasts (matched-pairs *t* tests, two-tailed) were the more demanding, they were adopted as the basis for sample-size determination and formed the preregistered basis for Experiment 2. For Experiment 1, based on effect sizes from prior block-design pupillometry studies^27^^,^^31^^,^^58^^,^^59^ (Cohen’s *d*z = 0.8) at a Bonferroni-corrected α of 0.0167 with 80% power, a minimum of 20 participants was required; for Experiments 2 and 3, based on the main effect observed in Experiment 1 (Cohen’s *d* = 0.69 as preregistered, No-thought vs. Inner speech) at the same corrected α, 25 participants were required. Recruitment targets, which included an allowance for attrition, were set at 25 (Experiment 1) and 30 (Experiments 2 and 3). For reference, the omnibus repeated-measures ANOVA (F tests; ANOVA: repeated measures, within factors), assuming a large effect (effect size *f* = 0.40), α = 0.05, power = 0.80, one group, three measurements, a correlation among repeated measures of 0.5, and a nonsphericity correction ε = 1, required a smaller total sample size of 12 (actual power = 0.82).

#### Exclusion criteria

Participants with insufficient valid pupil data were excluded from analysis: after 1-s binning, those with ≥50% missing samples across the session were removed, as were participants for whom valid pupil data were entirely unavailable for any one condition. These criteria led to the exclusion of two participants in Experiment 1, three in Experiment 2, and zero in Experiment 3.

#### Primary pupillometry analysis

For each experiment, a one-way repeated-measures ANOVA with condition (no-thought, inner speech, overt speech) as a within-subject factor and Δpupil diameter as the dependent variable tested whether conditions differed overall; degrees of freedom were corrected for violations of sphericity using the Greenhouse–Geisser correction. When a significant main effect was observed, pairwise comparisons used Bonferroni-corrected paired *t*-tests across three planned contrasts (no-thought vs. inner speech; inner speech vs. overt speech; no-thought vs. overt speech). One-sample *t*-tests against zero determined whether each condition’s Δpupil diameter deviated from the pre-task baseline. Effect sizes are reported as partial *η*^*2*^ for the repeated-measures ANOVAs and as Cohen’s *d*z for the paired and one-sample *t*-tests. The significance threshold was *p* < 0.05 throughout, and all analyses were performed in GraphPad Prism 9. The analytic approach was established in Experiment 1, preregistered for Experiment 2 prior to data collection, and applied without modification, and thus without preregistration, in Experiment 3. In figures, data are shown as mean ± SEM; “n” denotes the number of participants. Asterisks in the figures denote the significance of Bonferroni-corrected post hoc comparisons (∗*p* < 0.05, ∗∗*p* < 0.01, ∗∗∗*p* < 0.001).

#### Compliance and supplementary analyses

To verify compliance with the inner-speech instructions, Spearman’s *rho* was computed between the overt- and inner-speech final counts across participants in each experiment; a strong positive correlation would indicate that individual counting tempo was preserved across conditions. A paired *t* test additionally assessed whether the mean final count differed between the two conditions. As supplementary analyses, the single no-thought rating was summarized with descriptive statistics, and absolute (baseline) pupil diameter was compared across experiments to assess whether differences in initial pupil state contributed to the findings; both are reported in Data S1 and Figure S1.

### Additional resources

Experiment 1 was conducted first without preregistration but was hypothesis-driven rather than exploratory, testing *a priori* predictions about pupil-linked arousal and establishing the paradigm and analysis approach. Building on Experiment 1, Experiment 2 was then preregistered on the Open Science Framework (https://osf.io/7efbv) and conducted accordingly. Experiment 3 was identical to Experiment 2 in its sample-size rationale, design, and analysis framework, differing only in the counting task used to increase cognitive load, and was therefore not separately preregistered. Apart from this manipulation, all analysis procedures, including the sample-size determination, missing-data exclusion, data computation, measurement settings, and session duration, were identical to Experiment 1 and 2. Experiment 3 was also conducted after the preregistration of Experiment 2 and Experiment 2 data collection, so that its procedures followed the preregistered protocol of Experiment 2.

## References

[1] Geva S., Langland-Hassan P., Vicente A. (2018). Inner Speech: New Voices.

[2] Geva S., Jones P.S., Crinion J.T., Price C.J., Baron J.-C., Warburton E.A. (2011). The neural correlates of inner speech defined by voxel-based lesion-symptom mapping. Brain.

[3] Perrone-Bertolotti M., Rapin L., Lachaux J.P., Baciu M., Lœvenbruck H. (2014). What is that little voice inside my head? Inner speech phenomenology, its role in cognitive performance, and its relation to self-monitoring. Behav. Brain Res..

[4] Alderson-Day B., Fernyhough C. (2015). Inner speech: Development, cognitive functions, phenomenology, and neurobiology. Psychol. Bull..

[5] Price C.J. (2012). A review and synthesis of the first 20 years of PET and fMRI studies of heard speech, spoken language and reading. Neuroimage.

[6] Stephan F., Saalbach H., Rossi S. (2020). The brain differentially prepares inner and overt speech production: Electrophysiological and vascular evidence. Brain Sci..

[7] Zhang W., Jiang M., Teo K.A.C., Bhuvanakantham R., Fong L., Sim W.K.J., Guo Z., Foo C.H.V., Chua R.H.J., Padmanabhan P. (2024). Revealing the spatiotemporal brain dynamics of covert speech compared with overt speech: A simultaneous EEG-fMRI study. Neuroimage.

[8] Tian X., Poeppel D. (2012). Mental imagery of speech: linking motor and perceptual systems through internal simulation and estimation. Front. Hum. Neurosci..

[9] Whitford T.J., Jack B.N., Pearson D., Griffiths O., Luque D., Harris A.W., Spencer K.M., Le Pelley M.E. (2017). Neurophysiological evidence of efference copies to inner speech. eLife.

[10] Grandchamp R., Rapin L., Perrone-Bertolotti M., Pichat C., Haldin C., Cousin E., Lachaux J.-P., Dohen M., Perrier P., Garnier M. (2019). The ConDialInt model: Condensation, dialogality, and intentionality dimensions of inner speech within a hierarchical predictive control framework. Front. Psychol..

[11] Alderson-Day B., Weis S., McCarthy-Jones S., Moseley P., Smailes D., Fernyhough C. (2016). The brain’s conversation with itself: neural substrates of dialogic inner speech. Soc. Cognit. Affect Neurosci..

[12] Hurlburt R.T., Alderson-Day B., Kühn S., Fernyhough C. (2016). Exploring the ecological validity of thinking on demand: Neural correlates of elicited vs. Spontaneously occurring inner speech. PLoS One.

[13] Raut R.V., Rosenthal Z.P., Wang X., Miao H., Zhang Z., Lee J.-M., Raichle M.E., Bauer A.Q., Brunton S.L., Brunton B.W., Kutz J.N. (2025). Arousal as a universal embedding for spatiotemporal brain dynamics. Nature.

[14] Mäki-Marttunen V. (2021). Pupil-based states of brain integration across cognitive states. Neuroscience.

[15] Aston-Jones G., Cohen J.D. (2005). An integrative theory of locus coeruleus-norepinephrine function: adaptive gain and optimal performance. Annu. Rev. Neurosci..

[16] Cools R., Arnsten A.F.T. (2022). Neuromodulation of prefrontal cortex cognitive function in primates: the powerful roles of monoamines and acetylcholine. Neuropsychopharmacology.

[17] McGinley M.J., David S.V., McCormick D.A. (2015). Cortical membrane potential signature of optimal states for sensory signal detection. Neuron.

[18] Lin P.-A., Asinof S.K., Edwards N.J., Isaacson J.S. (2019). Arousal regulates frequency tuning in primary auditory cortex. Proc. Natl. Acad. Sci. USA.

[19] Dávila G., Torres-Prioris M.J., López-Barroso D., Berthier M.L. (2023). Turning the spotlight to cholinergic pharmacotherapy of the human language system. CNS Drugs.

[20] Joshi S., Gold J.I. (2020). Pupil size as a window on neural substrates of cognition. Trends Cognit. Sci..

[21] Schwalm M., Rosales Jubal E. (2017). Back to pupillometry: How cortical network state fluctuations tracked by pupil dynamics could explain neural signal variability in human cognitive neuroscience. eNeuro.

[22] Nobukawa S., Shirama A., Takahashi T., Toda S. (2024). Recent trends in multiple metrics and multimodal analysis for neural activity and pupillometry. Front. Neurol..

[23] Kuwamizu R., Grujic N., Minami T., Zou L., Ji L., Herold F., Chen A. (2026). The Handbook of Physical Activity and Cognitive Neuroscience.

[24] Reimer J., McGinley M.J., Liu Y., Rodenkirch C., Wang Q., McCormick D.A., Tolias A.S. (2016). Pupil fluctuations track rapid changes in adrenergic and cholinergic activity in cortex. Nat. Commun..

[25] Grujic N., Polania R., Burdakov D. (2024). Neurobehavioral meaning of pupil size. Neuron.

[26] Kuwamizu R., Yamazaki Y., Suwabe K., Suzuki K., Sankai Y., Soya H., Soya H. (2025). Exercise Brain Stimulation for Cognitive Function and Mental Health. Advances in Neurobiology.

[27] Yamazaki Y., Suwabe K., Nagano-Saito A., Saotome K., Kuwamizu R., Hiraga T., Torma F., Suzuki K., Sankai Y., Yassa M.A., Soya H. (2023). A possible contribution of the locus coeruleus to arousal enhancement with mild exercise: evidence from pupillometry and neuromelanin imaging. Cereb. Cortex Commun..

[28] Strauch C., Wang C.-A., Einhäuser W., Van der Stigchel S., Naber M. (2022). Pupillometry as an integrated readout of distinct attentional networks. Trends Neurosci..

[29] Kahneman D., Beatty J. (1966). Pupil diameter and load on memory. Science.

[30] Schaefer M., Mathôt S., Lundqvist M., Lundström J.N., Arshamian A. (2025). The pupillary respiratory-phase response: pupil size is smallest around inhalation onset and largest during exhalation. J. Physiol..

[31] Kuwamizu R., Yamazaki Y., Aoike N., Ochi G., Suwabe K., Soya H. (2022). Pupil-linked arousal with very light exercise: pattern of pupil dilation during graded exercise. J. Physiol. Sci..

[32] Hayashi N., Someya N., Fukuba Y. (2010). Effect of intensity of dynamic exercise on pupil diameter in humans. J. Physiol. Anthropol..

[33] Hess E.H., Polt J.M. (1964). Pupil size in relation to mental activity during simple problem-solving. Science.

[34] Koelewijn T., Zekveld A.A., Festen J.M., Kramer S.E. (2012). Pupil dilation uncovers extra listening effort in the presence of a single-talker masker. Ear Hear..

[35] Zekveld A.A., Koelewijn T., Kramer S.E. (2018). The pupil dilation response to auditory stimuli: Current state of knowledge. Trends Hear..

[36] Schwartz Z.P., Buran B.N., David S.V. (2020). Pupil-associated states modulate excitability but not stimulus selectivity in primary auditory cortex. J. Neurophysiol..

[37] Kaufman K.J., Krall R.F., Williamson R.S. (2026). Pupil-linked arousal heterogeneously modulates cell-type-specific sensory processing. Sci. Adv..

[38] Franke K., Willeke K.F., Ponder K., Galdamez M., Zhou N., Muhammad T., Patel S., Froudarakis E., Reimer J., Sinz F.H., Tolias A.S. (2022). State-dependent pupil dilation rapidly shifts visual feature selectivity. Nature.

[39] Takahashi T., Takikawa Y., Kawagoe R., Shibuya S., Iwano T., Kitazawa S. (2011). Influence of skin blood flow on near-infrared spectroscopy signals measured on the forehead during a verbal fluency task. Neuroimage.

[40] Fawcett J.M., Roberts B.R.T., Willoughby H.V., Tiller J.C., Hourihan K.L., MacLeod C.M. (2026). The pupillometric production effect: Evidence for enhanced processing preceding, during, and following production. Cognition.

[41] Brych M., Händel B.F., Riechelmann E., Pieczykolan A., Huestegge L. (2021). Effects of vocal demands on pupil dilation. Psychophysiology.

[42] Nalborczyk L., Debarnot U., Longcamp M., Guillot A., Alario F.-X. (2022). The role of motor inhibition during covert speech production. Front. Hum. Neurosci..

[43] Rozado D., Lochner M., Engelke U., Dünser A. (2019). Detecting intention through motor-imagery-triggered pupil dilations. Hum.-Comput. Interact..

[44] Voudouris D., Schuetz I., Schinke T., Fiehler K. (2023). Pupil dilation scales with movement distance of real but not of imagined reaching movements. J. Neurophysiol..

[45] Otani K., Watanabe R., Kuwamizu R., Yamamoto N., Moriguchi Y. (2026). The developmental origin of inner speech: Evidence from a mental counting task in 3- to 6-year-old children. Acta Psychol..

[46] Feynman R.P. (1988). It’s as simple as one, two, three. Eng. Sci..

[47] Tilsen S. (2024). Internal speech is faster than external speech: Evidence for feedback-based temporal control. Cognition.

[48] Joshi S., Li Y., Kalwani R.M., Gold J.I. (2016). Relationships between pupil diameter and neuronal activity in the locus coeruleus, colliculi, and cingulate cortex. Neuron.

[49] Grujic N., Tesmer A., Bracey E., Peleg-Raibstein D., Burdakov D. (2023). Control and coding of pupil size by hypothalamic orexin neurons. Nat. Neurosci..

[50] Papesh M.H., Goldinger S.D. (2012). Pupil-BLAH-metry: cognitive effort in speech planning reflected by pupil dilation. Atten. Percept. Psychophys..

[51] Kang O., Banaji M.R. (2020). Pupillometric decoding of high-level musical imagery. Conscious. Cognit..

[52] Dahl M.J., Mather M., Werkle-Bergner M. (2022). Noradrenergic modulation of rhythmic neural activity shapes selective attention. Trends Cognit. Sci..

[53] Schneider D.M., Nelson A., Mooney R. (2014). A synaptic and circuit basis for corollary discharge in the auditory cortex. Nature.

[54] Fernyhough C., Borghi A.M. (2023). Inner speech as language process and cognitive tool. Trends Cognit. Sci..

[55] Harvey A.G., Payne S. (2002). The management of unwanted pre-sleep thoughts in insomnia: distraction with imagery versus general distraction. Behav. Res. Ther..

[56] Moffatt J., Mitrenga K.J., Alderson-Day B., Moseley P., Fernyhough C. (2020). Inner experience differs in rumination and distraction without a change in electromyographical correlates of inner speech. PLoS One.

[57] Faul F., Erdfelder E., Lang A.-G., Buchner A. (2007). G∗Power 3: A flexible statistical power analysis program for the social, behavioral, and biomedical sciences. Behav. Res. Methods.

[58] Kuwamizu R., Yamazaki Y., Aoike N., Hiraga T., Hata T., Yassa M.A., Soya H. (2025). Limited potential for fNIRS photobiomodulation in exercise-induced pupil dynamics and improved executive function. Neuroimage.

[59] Kuwamizu R., Yamazaki Y., Aoike N., Hiraga T., Hata T., Yassa M.A., Soya H. (2023). Pupil dynamics during very light exercise predict benefits to prefrontal cognition. Neuroimage.

