# Supplementary material for: Dissociable pupil-linked arousal during overt and inner speech
Source: iScience. 2026 Oct 9;29(11):117825. doi: 10.1016/j.isci.2026.117825 (PMC13677175; doi:10.1016/j.isci.2026.117825)
Supplement: Supplementary file 1 [file mmc1.pdf]

iScience, Volume *29*

Supplemental information

Dissociable pupil-linked arousal during overt and inner speech

Ryuta Kuwamizu, Ryoichi Watanabe, Nozomi Yamamoto, Kota Otani, and Yusuke Moriguchi

Supplemental Information

Figure S1: Baseline pupil diameter across experiments, related to Figure 3.

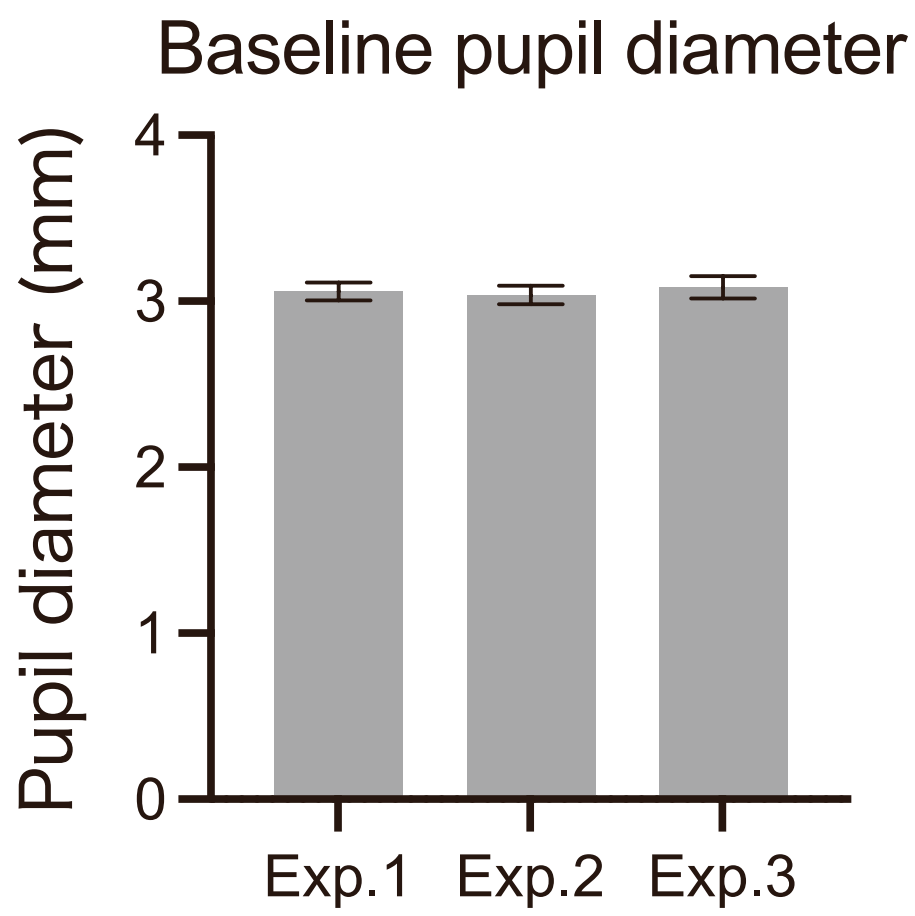

Absolute (unadjusted) baseline pupil diameter in Experiments 1, 2, and 3 (mean, SEM). To assess whether differences in initial pupil state could have contributed to the divergent baseline-related and no-thought findings across experiments, absolute (unadjusted) baseline pupil diameter, defined as the mean pupil diameter during the last 15 s of the rest period, was compared across the three experiments using a one-way between-subjects ANOVA. Baseline pupil diameter did not differ significantly across experiments ($F(2, 77) = 0.15$, $p = .860$, $\eta^2 = .004$), indicating that the current findings are unlikely to reflect differences in initial pupil state. Consistent with this, our main conclusions rest on within-experiment comparisons between the inner- and overt-speech conditions rather than on absolute baseline values across experiments.

Figure S2: Pupil responses across step-counting conditions in Experiment 3, related to Figure 3.

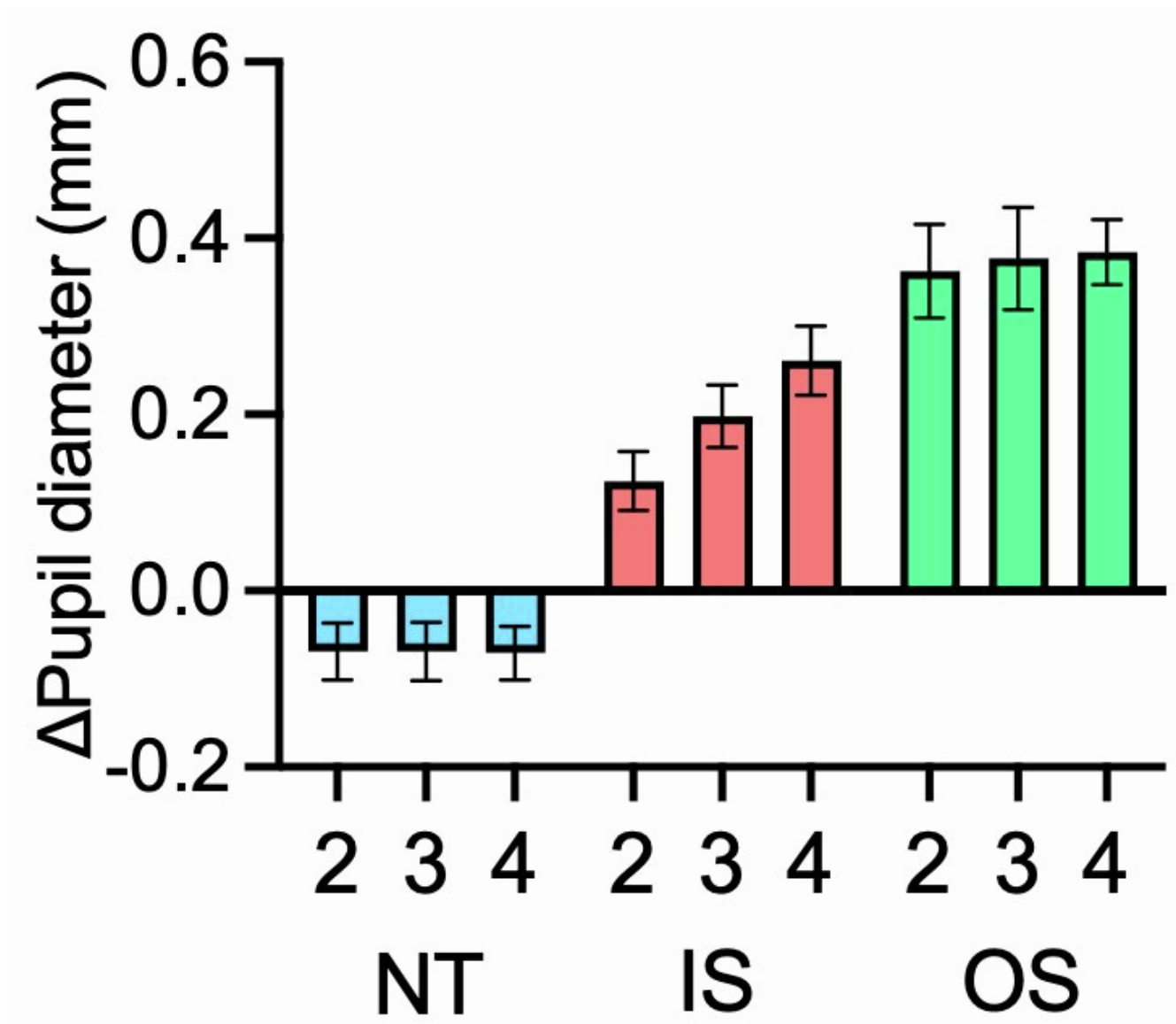

Change in pupil diameter (ΔPupil diameter, mm; mean ± SEM) in Experiment 3 for the no-thought (NT), inner-speech (IS), and overt-speech (OS) conditions, shown separately for the three step-counting conditions (steps of 2, 3, and 4). In Experiment 3, the three speech conditions were each tested under three step-counting conditions (counting in steps of 2, 3, and 4). Because pupil responses did not differ across these step-counting conditions, we averaged across them for the main analyses rather than analyzing them separately. A two-way repeated-measures ANOVA (speech condition × step-counting condition; Greenhouse–Geisser corrected; $n = 30$) confirmed this: a large main effect of speech condition ($F(1.57, 45.5) = 55.30$, $p < .0001$), no significant main effect of step-counting condition ($F(1.62, 47.0) = 1.79$, $p = .183$), and no significant speech × counting interaction ($F(3.22, 93.3) = 1.53$, $p = .210$). Exploratorily, however, within the IS condition pupil dilation increased with step size (one-way repeated-measures ANOVA: $F(1.96, 56.7) = 5.08$, $p = .0098$), being smallest at a step of 2 and largest at a step of 4. Although the overall interaction was not significant, this graded, step-dependent pattern is consistent with a load-dependent pupil-dilation effect during inner speech, in line with the higher-load rationale of Experiment 3.

Data S1: No-thought ratings across experiments.

In the no-thought condition, participants rated on a 0–10 scale (0 = thought about nothing at all; 10 = thought about various things) the degree to which they had been thinking about something; lower values indicate better maintenance of the no-thought state. The rating was collected once after the session in Experiment 1 (available for 20 of the 23 participants) and after each block, then averaged within participants, in Experiments 2 and 3.

Ratings were low throughout: Experiment 1, mean 3.75, median 3.00 (25th–75th percentile, 2.00–4.75); Experiment 2, mean 3.40, median 3.33 (2.67–3.67); Experiment 3, mean 2.99, median 2.33 (1.67–4.08), indicating that participants reported relatively little thought during the condition.
